# Haematological and biochemical reference intervals for free-ranging brown bears (*Ursus arctos*) in Sweden

**DOI:** 10.1186/s12917-014-0183-x

**Published:** 2014-08-21

**Authors:** Anne Randi Græsli, Åsa Fahlman, Alina L Evans, Mads Frost Bertelsen, Jon Martin Arnemo, Søren Saxmose Nielsen

**Affiliations:** 1 Department of Large Animal Sciences, University of Copenhagen, Grønnegårdsvej 8, Frederiksberg C, DK-1870, Denmark; 2 Center for Zoo and Wild Animal Health, Copenhagen Zoo, Roskildevej 38, Frederiksberg, DK-2000, Denmark; 3 Department of Forestry and Wildlife Management, Faculty of Applied Ecology and Agricultural Sciences, Hedmark University College, Campus Evenstad, Elverum, NO-2418, Norway; 4 Department of Clinical Sciences, Faculty of Veterinary Medicine and Animal Science, Swedish University of Agricultural Sciences, Uppsala, SE-750 07, Sweden; 5 Department of Wildlife, Fish and Environmental Studies, Faculty of Forest Sciences, Swedish University of Agricultural Sciences, Umeå, SE-901 83, Sweden

**Keywords:** Brown bear, Ursus arctos, Haematology, Biochemistry, Reference intervals

## Abstract

**Background:**

Establishment of haematological and biochemical reference intervals is important to assess health of animals on individual and population level. Reference intervals for 13 haematological and 34 biochemical variables were established based on 88 apparently healthy free-ranging brown bears (39 males and 49 females) in Sweden. The animals were chemically immobilised by darting from a helicopter with a combination of medetomidine, tiletamine and zolazepam in April and May 2006–2012 in the county of Dalarna, Sweden. Venous blood samples were collected during anaesthesia for radio collaring and marking for ecological studies. For each of the variables, the reference interval was described based on the 95% confidence interval, and differences due to host characteristics sex and age were included if detected. To our knowledge, this is the first report of reference intervals for free-ranging brown bears in Sweden.

**Results:**

The following variables were not affected by host characteristics: red blood cell, white blood cell, monocyte and platelet count, alanine transaminase, amylase, bilirubin, free fatty acids, glucose, calcium, chloride, potassium, and cortisol. Age differences were seen for the majority of the haematological variables, whereas sex influenced only mean corpuscular haemoglobin concentration, aspartate aminotransferase, lipase, lactate dehydrogenase, β-globulin, bile acids, triglycerides and sodium.

**Conclusions:**

The biochemical and haematological reference intervals provided and the differences due to host factors age and gender can be useful for evaluation of health status in free-ranging European brown bears.

## Background

Establishment of haematological and biochemical reference intervals is an important tool to assess health of animals and understand the impact of disease on individual and population levels [1],[2]. The brown bear (*Ursus arctos*), is the only member of the *Ursidae* native to the Scandinavian mainland [3]. In Sweden, most brown bears inhabit the northern 2/3 of the country. An estimation of the population size in Sweden in 2008 was 3,298 individuals (95% confidence intervals; 2,968 - 3,667) [4].

Various factors may affect the biochemical and haematological variables and should be considered when establishing and using reference intervals. Extrinsic factors include factors that may stress the animal, whereas intrinsic factors are associated with host characteristics.

Only few studies have evaluated biochemistry and haematology in European brown bears [5]-[12], and the published data are mostly based on low numbers of animals or specimen from captive bears, both of which may not be representative of brown bears in general. The objectives of this study were to establish reference intervals for standard haematological and biochemical variables in free-ranging brown bears immobilised with medetomidine-tiletamine-zolazepam in Sweden in the spring (April and May), and to determine if the host characteristics age and sex influenced the haematological and biochemical variables.

## Methods

### Study site and animals

The study area was in the county of Dalarna, Sweden, approximately 61°N, 15°E, at altitudes of 300–700 m above sea level. The study included 88 free-ranging brown bears (39 males and 49 females), anaesthetised in April and May 2006 to 2012. Venous blood samples were collected during anaesthesia for radio collaring and marking for ecological studies. All bears were darted from a helicopter with a combination of medetomidine, tiletamine and zolazepam. The capture procedure is described in detail elsewhere [13]. All bears appeared clinically healthy as determined by clinical examination during anaesthesia. The bears were between 1 and 18 years of age. For animals that had not been followed from birth, age determination was performed by evaluation of an extracted rudimentary first maxillary premolar tooth [14]. Age was grouped into three categories: yearling (15 months), subadult (2–4 years old) and adult (≥5 years old). Fourteen bears were resampled during multiple years; in these cases, only one sample from each bear was included and randomly selected using a random number generator. A total of 69 samples for haematology and 88 samples for clinical chemistry were analysed. The capture and sampling were approved by the Ethical Committee on Animal Experiments, Uppsala, Sweden (application numbers: C 7/12, C 47/9 and C 59/6).

### Sampling procedure

Blood was collected from the jugular vein using a vacutainer system (BD Vacutainer®, BD Diagnostics, Preanalytical Systems, Franklin Lakes, NJ, USA). Blood was sampled approximately 60 minutes after darting. Blood for haematological analyses was collected in 4 mL tubes, with EDTA (ethylenediaminetetraacetic acid) as anticoagulant (Vacuette®, Greiner Bio-One International AG, Kremsmünster, Austria). Two slides with blood smears, from each bear, were performed just after collection. The blood was kept refrigerated until shipment, and kept cool by cooling elements during the transport to the Clinical Chemistry Laboratory at the Swedish University of Agricultural Sciences, Uppsala, Sweden. The time from sampling to analysis was two days. After arrival to the laboratory, the blood slides were stained with May–Grünwald–Giemsa stain.

Blood for biochemistry was collected in 9 mL tubes with gel and clot activating factor (Vacuette®, Greiner Bio-One International AG, Kremsmünster, Austria). The blood tubes were kept at room temperature for 1–2 hours to ensure complete clotting, and then centrifuged at 1500 × g for 10 minutes to separate the serum. The serum was stored in 2 mL cryogenic vials (Nalgene, Nalge Company, Rochester, NY, USA) and kept at −20°C until shipment to the Central Laboratory, Norwegian School of Veterinary Science, Oslo, Norway. The blood tubes were kept cool by ice packs during shipment, and stored at −80°C until analysis.

### Laboratory analyses

The haematology profile included red blood cell (RBC) count, white blood cell (WBC) count, haemoglobin, haematocrit, mean corpuscular volume (MCV), mean corpuscular haemoglobin concentration (MCHC) and platelet count. The Cell-Dyn 3500 Hematology Analyser (Abbott Laboratories, Abbott Park, Illinois, USA) was used for the haematological analyses in 2006 and 2007. From 2008, the ADVIA®2120 Hematology System was used (Bayer HealthCare, Diagnostics Division, Tarrytown, NY, USA). In addition, a white blood cell differential count (percentage of total) for band shaped neutrophils, segmented neutrophils, eosinophils, basophils, lymphocytes and monocytes, was carried out by manual examination of a blood smear, performed by the same technician. The technician counted 100 white blood cells.

The biochemical profile included aspartate aminotransferase (AST), alanine transaminase (ALT), alkaline phosphatase (AP), γ-glutamyl transpeptidase (GGT), glutamate dehydrogenase (GD), creatinine kinase (CK), lactate dehydrogenase (LD), amylase, lipase, total protein, urea, creatinine, uric acid, bile acids, total bilirubin, cholesterol, triglycerides, free fatty acids (FFA), β-hydroxybutyrate (β-HBA), glucose, inorganic phosphorous, calcium, magnesium, sodium, potassium, chloride, iron, and cortisol. Serum protein electrophoresis yielded five protein fractions; albumin, α_1_-globulin, α_2_-globulin, β-globulin, γ-globulin, and the albumin-globulin ratio. All clinical chemistry analyses, except cortisol analysis, were carried out with an ADVIA®1650 Chemistry System (Siemens Healthcare, Tarrytown, NY, USA) from 2006 to 2011. Thereafter it was replaced by the ADVIA®1800 Chemistry System (Siemens Healthcare). The analytical methods used for the analysis are given in Table 1. Cortisol was measured with an Immulite system (Diagnostic Products Corporation, Los Angeles, CA, USA) and the protein fractions were measured by serum protein electrophoresis. Reagents from Siemens Medical Solutions Diagnostics were used for all analytes except FFA (Wako Chemicals GmbH, Neuss, Germany), β-HBA and GD (Randox Laboratories Ltd., Dublin, Ireland) [15].

**Table 1:** Analytical methods used in ADVIA®1650 and ADVIA®1800 Chemistry Systems for blood analysis from brown bears

**Analytes**	**Analytical concepts**
ALT	Modified IFCC
Amylase	Ethylidene blocked-pNPG7
AP	Modified IFCC
AST	Modified IFCC
β-HBA	Enzymatic/NAD + −NADH
Total bilirubin	Vanadate oxidation
Inorganic phosphorus	Phosphomolybdate/UV
FFA	Enzymatic-colorimetric
Bile acids	Enzymatic amplification/Thio-NAD-Thio-NADH
GGT	Modified IFCC
Glucose	Hexokinase
GD	DGKC/NADH-NAD+
Iron	Ferrozine
Calcium	CPC
Potassium	Ion selective electrode (ISE), diluted
Chloride	Ion selective electrode (ISE), diluted
Cholesterol	Enzymatic/trinder
CK	NAC activated (DGKC)
Creatinine	Jaffe, kinetic
LD	Enzymatio (L-P) / TRIS / NAD + −NADH
Lipase	Colorimetric rate
Magnesium	Xylidyl blue
Sodium	Ion selective electrode (ISE), diluted
Total Protein	Biuret
Triglycerides	Enzymatic/trinder without serum blank
Urea	Enzymatic: urease with GLDH
Uric Acid	Enzymatic/trinder

### Statistical analysis

The guidelines from the American Society for Veterinary Clinical Pathology’s Quality Assurance and Laboratory Standard Committee were used for the establishment of reference intervals in this study [2]. To establish reference intervals for the biochemical and haematological variables, and to determine if these should be stratified based on age and sex, the following strategy was used: haematological and biochemical variables were included as continuous variables and their distributions determined. Descriptive statistics are presented with upper and lower 95% confidence interval for all variables, and mean and standard deviation for variables that were normally distributed. The distributions of the haematological and biochemical variables were assessed using histograms and QQ-plots, including an assessment if they had a Normal distribution. Those with an approximate Normal distribution were kept on the direct scale, whereas Box-Cox transformation was used for variables that did not appear with a Normal distribution of the direct scale.

The formula for the Box-Cox transformation, where $\bar{y}$ is the geometric mean [16];

(1) $$\begin{array}{l} y{'}_{\lambda }=\frac{y^{{}_{\lambda }}-1}{\lambda \cdot {\bar{y}}^{\lambda -1}},\mathrm{for}\;\lambda \neq 0,\;\mathrm{and}\\ y{'}_{\lambda }=\bar{y}\cdot \log\;\left(y\right),\mathrm{for}\;\lambda =0, \end{array}$$

When >90% of the values for a given variable were 0, transformation was deemed non-sensible. Therefore the reference intervals for these variables are presented the 90^th^, 97.5^th^ and 100^th^ percentiles. For variables including observed values of 0, a factor of 0.05 or 0.5 was added to all the values before transformation to avoid impossible mathematical operations. In case a transformation was used, the transformation parameter λ was reported.

Comparisons between age and sex were performed on the transformed variables using analysis of variance. A significant *F*-test was followed by a simultaneous comparison of paired means using the Tukey-Kramer test, to evaluate if there was a significant difference between age groups and genders. After calculating the reference intervals, the transformed data were back-transformed to the original scale. The added values of 0.05 or 0.5 were subtracted from the variables where they had been added. Resulting reference intervals were presented in sex and age cohorts if significantly different or otherwise combined. Data were presented as 95% confidence intervals.

Statistical analyses were performed using JMP®10.0.0 statistical software (SAS Institute, Cary, North Carolina, USA), and a *p*-value <0.05 was considered significant.

## Results

Sixty-nine haematological specimens (44 females and 25 males) were included in the statistical analysis. These were from 23 adults, 19 subadults and 27 yearlings. In 21 of the specimens there was platelet aggregation, so because of the small sample size in each group, the variable platelet count was not included in the analysis of variance. As >90% of the values obtained for band-shaped neutrophils and basophils were 0, they were presented as 90^th^, 97.5^th^ and 100^th^ percentiles, which were 4.0; 15.3 and 19.0; 0.0, 2.0 and 2.3, for band-shaped neutrophils and basophils, respectively. Eighty-eight biochemical specimens (49 females and 39 males) were included in the statistical analysis. They were from 36 adults, 25 subadults and 27 yearlings. Reference intervals for haematological and biochemical variables for bears in the spring are presented in Tables 2, 3, 4 and 5, also including λ-values from the Box-Cox transformations and the 2.5^th^ and 97.5^th^ percentiles on untransformed data.

**Table 2:** **Haematological reference intervals for brown bears (*Ursus arctos*) in Sweden presented as 95% confidence interval**^**1**^

**Variable**^**a**^	**Unit**	***n***	**λ**^**b**^	**2.5 - 97.5 interval**^**c**^	**Sex**^**d**^	**Age group**^**e**^	**Reference interval**	***p*-value**^**f**^
Haemoglobin	g/L	69	1.8	142-189	B	Y	137-171	<0.0001^A^
						SA	154-188	
RBC	×10^12^/L	69	0	6.2-8.0	B	YSA	6.2-8.0	
Haematocrit	L/L	69	0.6	0.41-0.54	B	Y	0.40-0.49	<0.0001^A^
						SA	0.43-0.54	
MCV	fL	69	2	57-75	B	Y	57-70	<0.0001^A^
						SA	63-75	
MCHC	g/L	69	0	334-373	F	YSA	332-366	0.0062^AxS^
					M	Y	332-366	
						SA	350-374	
WBC	×10^9^/L	69	−0.2	3.6-11.4	B	YSA	3.9-11.5	
Segmented neutrophils	%	69	2	57.8-92.5	B	Y	62.2-93.6	0.0185^A^
						SA	58.4-85.9	
Eosinophils	%	69	0	0.0-9.6	B	Y	0.0-4.3	<0.0001^A^
						SA	0.0-11.1	
Lymphocytes	%	69	0.4	2.5-32.5	B	YS	4.8-32.6	0.001^A^
						A	1.0-27.7	
Monocytes	%	69	0.2	0.8-11.3	B	YSA	0.7-11.7	
Platelet count	×10^9^/L	48	0.4	103-811	B	YSA	141-520	

^1^: Presented stratified by age group, sex or presented overall, depending on if a difference between groups was detected. ^a^: RBC=red blood cell count; MCV=mean corpuscular volume; MCHC=mean corpuscular haemoglobin concentration; WBC=white blood cell count. ^b^: λ=the transformation value from the Box-Cox transformation. ^c^: 2.5 and 97.5 percentiles on untransformed data. ^d^: B=both female and male; F=female; M=male. ^e^: Y=yearling; S=subadult; A=adult. ^f^: *p*-values are given for significantly different groups: ^A^=*p*-value for age and ^AxS^=*p*-value for age-sex interaction.

**Table 3:** **Enzyme reference intervals for brown bears (*Ursus arctos*) presented as 95% confidence interval**^**1**^

**Variable**^**a**^	**Unit**	***n***	**λ**^**b**^	**2.5 - 97.5 interval**^**d**^	**Sex**^**c**^	**Age group**^**e**^	**Reference interval**	***p*-value**^**f**^
AST	U/L	88	−1.2	45-215	F	YA	56-168	0.0034^AxS^
						S	46-114	
					M	YSA	46-114	
ALT	U/L	88	−1	11-47	B	YSA	12-41	
AP	U/L	88	0.2	15-166	B	Y	65-185	<0.0001^A^
						S	31-101	
						A	10-92	
GGT	U/L	88	0.2	3-91	B	YS	3-51	0.0126^A^
						A	5-82	
GD	U/L	88	−0.8	2-18	B	YS	3-8	0.0003^A^
						A	3-16	
CK	U/L	88	−0.4	53-690	B	Y	81-631	<0.0001^A^
						SA	44-419	
Amylase	U/L	88	0	22-192	B	YSA	24-143	
Lipase	U/L	88	−0.4	14-247	F	YA	16-NE*	0.0063^A^, <0.0001^S^
						S	10-NE*	
					M	YA	11-159	
						S	9-58	
LD	U/L	88	0.2	434-1066	F	Y	587-1060	0.0184^AxS^
						SA	421-875	
					M	YS	587-1060	
						A	421-875	

^1^: Presented stratified by age group, sex or presented overall, depending on if a difference between groups was detected. ^a^: AST=aspartate aminotransferase; ALT=alanine transaminase; AP=alkaline phosphatase; GGT=γ-glutamyl transpeptidase; GD=glutamate dehydrogenase; CK=creatinine kinase; LD=lactate dehydrogenase; A:G=albumin:globulin ratio. ^b^: λ=the transformation value from the Box-Cox transformation. ^c^: 2.5 and 97.5 percentiles on untransformed data. ^d^: B=both female and male; F=female; M=male. ^e^: Y=yearling; S=subadult; A=adult. ^f^ : *p*-values are given for significantly different groups: ^A^=*p*-value for age, ^S^=*p*-value for sex and ^AxS^=*p*-value for age-sex interaction. *NE: Non-estimable because estimates were above the range of the transformation.

**Table 4:** **Metabolite and cortisol reference intervals for brown bears (*Ursus arctos*) presented as 95% confidence interval**^**1**^

**Variable**^**a**^	**Unit**	**n**	**λ**^**b**^	**2.5 - 97.5 interval**^**d**^	**Sex**^**c**^	**Age group**^**e**^	**Reference interval**	**p-value**^**f**^
β-HBA	mmol/L	88	−0.2	0.0-1.9	B	YSA	0.0-1.1	
Bile acids	μmol/L	88	−0.6	5-65	F	YA	8-59	0.0073^A^, 0.0059^S^
						S	5-60	
					M	YA	6-46	
						S	7-13	
Bilirubin (total)	μmol/L	88	0.2	0-6	B	YSA	0-5	
Cholesterol	mmol/L	88	1.2	4.5-12.2	B	Y	5.9-13.2	<0.0001^A^
						SA	4.7-10.4	
Creatinine	μmol/L	88	1	66-218	B	Y	57-160	<0.0001^A^
						S	101-190	
						A	107-235	
Free fatty acids	mmol/L	88	0.2	0.1-1.4	B	YSA	0.1-1.3	
Glucose	mmol/L	88	0.4	3.3-11.5	B	YSA	3.3-10.9	
Triglycerides	mmol/L	88	0.8	1.7-5.4	F	Y	1.5-5.4	0.0220^A^, 0.0122^S^
						SA	1.7-4.4	
					M	Y	3.2-8.6	
						SA	1.9-7.9	
Urea	mmol/L	88	−0.4	0.5-11.9	B	Y	0.4-4.0	0.0255^A^
						SA	0.4-NE*	
Uric acid	μmol/L	88	−0.8	68-209	B	Y	73-111	<0.0001^A^
						SA	70-222	
Cortisol	nmol/L	88	0.4	30-564	B	YSA	45-565	

^1^: Presented stratified by age group, sex or presented overall, depending on if a difference between groups was detected. ^a^: β-HBA=β-hydroxybutyrate ^b^: λ=the transformation value from the Box-Cox transformation. ^c^: 2.5 and 97.5 percentiles on untransformed data. ^d^: B=both female and male; F=female; M=male. ^e^: Y=yearling; S=subadult; A=adult. ^f^: *p*-values are given for significantly different groups: ^A^=*p*-value for age and ^S^=*p*-value for sex *NE: Non-estimable because estimates were above the range of the transformation.

**Table 5:** **Mineral and protein reference intervals for brown bears (*Ursus arctos*) presented as 95% confidence interval**^**1**^

**Variable**^**a**^	**Unit**	***n***	**λ**^**b**^	**2.5 - 97.5 interval**^**d**^	**Sex**^**c**^	**Age group**^**e**^	**Reference interval**	***p*-value**^**f**^
Calcium	mmol/L	88	2	1.8-2.7	B	YSA	1.8-2.6	
Chloride	mmol/L	88	2	88-104	B	YSA	92-105	
Iron	μmol/L	88	0.2	13-68	B	YS	14-50	<0.0001^A^
						A	19-75	
Magnesium	mmol/L	88	1	0.70-1.21	B	Y	0.70-1.27	0.0167^A^
						SA	0.65-1.15	
Inorganic Phosphorous	mmol/L	88	1.4	0.6-2.6	B	Y	1.1-2.7	0.0002^A^
						SA	0.7-2.3	
Potassium	mmol/L	88	−0.2	3.2-5.3	B	YSA	3.3-5.1	
Sodium	mmol/L	88	2	128-141	F	YSA	132-141	0.0135^S^
					M	YSA	123-145	
Albumin	g/L	86	1.4	27.2-49.6	B	Y	24.5-40.6	<0.0001^A^
						SA	34.2-49.1	
α_1_-globulin	g/L	86	0.4	2.6-7.9	B	Y	3.0-10.6	<0.0001^A^
						SA	1.7-8.3	
α_2_-globulin	g/L	86	−0.4	2.9-10.2	B	Y	3.1-13.4	0.0004^A^
						SA	2.7-9.3	
β-globulin	g/L	86	0	3.0-9.2	F	Y	3.2-6.0	<0.0001^A^, 0.0096^S^
						SA	3.7-9.1	
					M	Y	2.2-8.6	
						SA	5.1-10.0	
γ-globulins	g/L	86	0.4	1.6-7.9	B	Y	1.4-5.0	<0.0001^A^
						S	2.2-5.8	
						A	3.2-8.1	
A:G		86	0	1.13-2.89	B	Y	1.03-2.70	<0.0001^A^
						SA	1.38-3.07	
Total protein	g/L	88	2	48-72	B	Y	39-64	<0.0001^A^
						S	50-68	
						A	53-74	

^1^: Presented stratified by age group, sex or presented overall, depending on if a difference between groups was detected. ^a^: A:G=albumin:globulin ratio. ^b^: λ=the transformation value from the Box-Cox transformation. ^c^: 2.5 and 97.5 percentiles on untransformed data. ^d^: B=both female and male; F=female; M=male. ^e^: Y=yearling; S=subadult; A=adult. ^f^: *p*-values are given for significantly different groups: ^A^=*p*-value for age; ^S^=*p*-value for sex.

## Discussion

This study provides the first comprehensive report on reference intervals for free-ranging brown bears in Sweden. Variation caused by the capture and immobilisation procedure (stress, helicopter pursuit and anaesthetics), blood collection, and analytic procedures may affect the results, but are an inherent challenge in studies involving free ranging animals.

Both chasing prior to immobilisation and the anaesthetic drugs can potentially induce stress in addition to other factors such as fear, shock, pain and acid–base imbalance which can occur during immobilisation [17]. Stress increases the release of glucocorticoid hormones (cortisol and corticosterone) which results in increased gluconeogenesis and decreases use of glucose, decreased sensitivity to insulin, and fat and protein metabolism [18]. This results in increased blood glucose levels during stress. Plasma concentrations of cortisol have been used as an indicator of stress in different animals, but the correlation between visually evaluated amounts of handling stress and plasma cortisol levels are controversial [19]-[21]. The shift in the composition of white blood cells, known as a stress leukogram, characterised with a mild neutrophilia accompanied by a lymphopenia and eosinophilia and, less frequently with a monocytosis are well known [22]. The anaesthetics used can potentially affect the blood variables. Medetomidine has been shown to increase the blood glucose level in different species of animals [23]-[26]. A decrease in the total protein level is also reported after administration of medetomidine [23],[25]. The effect of medetomidine on cortisol levels in the blood of different animals is controversial, as some studies reports an increase, some a decrease and some reports that there is no change [24],[26]-[29]. To the authors’ knowledge, only few studies have looked at the effects of ketamine or tiletamine/zolazepam on blood variables. In dogs, the effect of ketamine has been linked to effects caused by stress, with increased plasma values of glucose and cortisol [30].

Lower haemoglobin, haematocrit and MCV in yearlings compared to subadult and adult animals are well described phenomena in brown bears [5],[12],[31]. Hypochromic microcytic anaemia in young, growing bears has been a suggested cause, attributable to iron deficiency caused by growth demands [31],[32]. Lower values of iron in yearlings and subadults compared to adults were found, but RBC counts were similar, so the yearlings in this study were not deemed to be anaemic. It is possible that the differences observed relate to differences in diet among the age groups [33].

The variation in white blood cell differential count in different age groups could reflect maturation of the immune system, because younger animals are known to have a higher lymphocyte count than adult animals [22]. The higher mean eosinophil levels in yearlings and subadults could potentially be explained by parasites in some individuals, however, this was not evaluated.

Alkaline phosphatase (AP) and inorganic phosphorous were highest in yearlings and lowest in adults, which probably reflected elevated levels in young growing animals due to increased osteoblastic activity during rapid bone growth [34]. Similar results for AP have been reported previously for other bears [6],[32],[35],[36] and for inorganic phosphorous in grizzly bears, black bears and wolves [6],[36],[37]. Only juvenile mammals are able to mobilise magnesium from the skeleton in the face of inadequate dietary intake, which could explain why younger bears had significantly higher serum magnesium concentrations than subadults and adults [38].

The higher values of lipase in females compared to males is in agreement with findings in polar bears [39]. Increased serum lipase activity could be associated to renal or pancreatic disease [34], while decreased serum amounts of lipase could be associated to lipaemic specimens [40]. However, none of the brown bear specimens from the present study, or the polar bear specimens in earlier mentioned study, were classified as lipaemic. Increased serum values of LD and AST could be a result of hepatocyte or muscle damage [34]. An intensive chase before immobilisation and dart trauma could induce acute skeletal muscle injury, and increased serum values.

Higher levels of total serum protein and albumin in older brown bears compared to younger brown bears have been previously described in other bear species [33],[35]. A correlation between primary food source and total protein levels has been described [33], and may explain the differences we found between yearlings and subadult and adult bears. Total serum protein and particularly albumin levels are indicators of dietary protein levels, so an increase from yearlings to subadults to adults could reflect better physiological condition in older bears [35]. The higher albumin:globulin ratio in subadult and adult bears compared to yearlings is also likely due to the higher level of albumin and total protein in the older bears. The increase of gamma globulins with increasing age may indicate a still developing antibody producing mechanism in younger individuals [6],[35],[41]. Similar differences have been demonstrated previously in brown bears and black bears [10],[35].

The higher values of cholesterol and triglycerides in yearlings compared to subadult and adult brown bears stand in contrast to a study of sloth bears, where the heaviest sloth bears had the highest values of cholesterol and triglycerides. This could be caused by an increased calorie intake or a decreased physical activity in the heaviest bears, or be due to an age related variation in diet [42]. The sloth bears were kept under semi-captive conditions with access to natural food resources. In studies of black bears, body fat mass correlated better with triglyceride and cholesterol values, than body weight. Significant differences in blood lipid concentrations among populations may reflect differences in diet [35],[43]. The higher cholesterol and triglyceride values in yearlings compared to subadult and adult brown bears in the present study could be a result of differences in diet in the age-groups, and a potential correlation between body fat mass and blood lipid concentrations would be an interesting avenue for further study.

The higher creatinine in adults compared to subadults, and in subadults compared to yearlings is in agreement with previous reports in bears [6],[36]. It has been concluded that the higher amounts of creatinine was due to larger muscle mass in older bears [6], although higher values would then be expected in males than in females, especially in adult bears, but this was not documented in the present study. Creatinine values have been reported to decrease during immobilisation, and the elevated levels in the first specimen could have been caused by exercise prior to the immobilisation [6].

Urea is a product of the protein metabolism, so differences in protein contents in the diet could be reflected in the blood values of urea. In early spring when the bears have left the den they need to increase their muscle mass, and protein rich food is the main energy source [44]. The lower urea values in yearlings may be due to different dietary preferences in yearlings.

A small sample size is often a limitation when sampling wild animals for research studies. Where significant differences among age groups or genders were detected, it was not possible to establish reference intervals. This was often because of a large standard error due to a small sample size and a large distribution of the values. The standard error could have been decreased with removal of outliers, but elimination of apparent outliers or unexpected values should not be done indiscriminately [2], and the large standard error might have been influenced by the preanalytical factors. Consequently, this could not really be addressed further except noting the presence of differences.

## Conclusion

To conclude, we provided haematological and biochemical reference intervals, which can be useful in health assessments of free-ranging brown bears. Differences in some host and ecological factors exist, which should be considered when interpreting diagnostic data.

## Competing interests

The authors have declared that they have no competing interests.

## Authors’ contributions

ARG analyzed the data and drafted the manuscript. JMA, ALE and ÅF conceived, designed and performed the experiments. SSN and MFB supervised data analysis. All authors participated in writing the manuscript and approved the final version.
